# Recovery of Forearm and Fine Digit Function After Chronic Spinal Cord Injury by Simultaneous Blockade of Inhibitory Matrix Chondroitin Sulfate Proteoglycan Production and the Receptor PTPσ

**DOI:** 10.1089/neu.2023.0117

**Published:** 2023-11-30

**Authors:** Adrianna J. Milton, Jessica C.F. Kwok, Jacob McClellan, Sabre G. Randall, Justin D. Lathia, Philippa M. Warren, Daniel J. Silver, Jerry Silver

**Affiliations:** ^1^ Department of Neurosciences, Case Western Reserve University, Cleveland, Ohio, USA.; ^2^ School of Biomedical Sciences, Faculty of Biological Sciences, University of Leeds, Leeds, United Kingdom.; ^3^ Institute of Experimental Medicine, Czech Academy of Science, Prague, Czech Republic.; ^4^ Department of Cardiovascular and Metabolic Sciences, Cleveland Clinic Lerner Research Institute, Cleveland, Ohio, USA.; ^5^ Department of Molecular Medicine, Cleveland Clinic Lerner College of Medicine at Case Western Reserve University, Cleveland, Ohio, USA.; ^6^ Wolfson Centre for Age-Related Diseases, King's College London, London, United Kingdom.

**Keywords:** axon regeneration, axon sprouting, CSPGs, perineuronal net, receptor PTPσ, spinal cord injury

## Abstract

Spinal cord injuries (SCI), for which there are limited effective treatments, result in enduring paralysis and hypoesthesia, in part because of the inhibitory microenvironment that develops and limits regeneration/sprouting, especially during chronic stages. Recently, we discovered that targeted enzymatic removal of the inhibitory chondroitin sulfate proteoglycan (CSPG) component of the extracellular and perineuronal net (PNN) matrix via Chondroitinase ABC (ChABC) rapidly restored robust respiratory function to the previously paralyzed hemi-diaphragm after remarkably long times post-injury (up to 1.5 years) following a cervical level 2 lateral hemi-transection. Importantly, ChABC treatment at cervical level 4 in this chronic model also elicited improvements in gross upper arm function. In the present study, we focused on arm and hand function, seeking to highlight and optimize crude as well as fine motor control of the forearm and digits at lengthy chronic stages post-injury. However, instead of using ChABC, we utilized a novel and more clinically relevant systemic combinatorial treatment strategy designed to simultaneously reduce and overcome inhibitory CSPGs. Following a 3-month upper cervical spinal hemi-lesion using adult female Sprague Dawley rats, we show that the combined treatment had a profound effect on functional recovery of the chronically paralyzed forelimb and paw, as well as on precision movements of the digits. The regenerative and immune system related events that we describe deepen our basic understanding of the crucial role of CSPG-mediated inhibition via the PTPσ receptor in constraining functional synaptic plasticity at lengthy time points following SCI, hopefully leading to clinically relevant translational benefits.

## Introduction

Each year, between 250,000 and 500,000 people worldwide suffer a debilitating spinal cord injury (SCI), with over half occurring at the cervical level, leading to millions chronically paralyzed as reported by the World Health Organization.^1^ Damage to the high cervical spinal cord can result in paralysis of much of the body, with devastating impacts on controlled, voluntary arm, hand, and digit movements, leading to a severe decrease in individual autonomy and overall quality of life.^2,3^ The last several decades of research have demonstrated the crucial role of the cell and axon growth inhibitory properties of chondroitin sulfate proteoglycans (CSPGs) during normal neural development as well as their inhibition of re-growth of cells or their processes after injury.^4–10^ In particular, we have focused on studying the role of the CSPG family of extracellular matrix molecules in the glial scar and the perineuronal net (PNN) as critical inhibitors of functional regeneration and sprouting after SCI.^8,10–14^

Within a few days after injury, CSPGs increase within and adjacent to the forming glial scar.^15–19^ Matrix upregulation also occurs immediately following SCI as part of a tightly regulated and crucial immune response, and the upregulation persists in the extracellular space and PNN in areas rostral and caudal to the injury site in denervated distal targets.^8,10–13,20^ Thus, inhibitory CSPGs upregulate expansively within and proximal and distal to the lesion epicenter, leading to limited regeneration through the lesion or back into target nuclei and curtailing potential sprouting/plasticity over large distances, which could occur from spared fiber systems either ipsilateral or contralateral to the lesion.^8,10–13,15,16,21,22^ The regulatory effects of CSPGs on both myelin and axon regeneration as well as neuronal plasticity after an acute^9,17,23–29^ or sub-chronic SCI,^5,17,23,26,30^ and in the context of many other conditions, have been well documented.^8,10,31–37^

The glycosaminoglycan (GAG) side chains, especially those containing 4-O-sulfated CS-E,^38,39^ are known to bind with highest affinity to their receptor protein tyrosine phosphatase PTPσ (rPTPσ)^38^ and provide much of the inhibitory properties of CSPGs.^39–41^ Their effects can be greatly decreased by enzymatic digestion using the bacterial enzyme Chondroitinase ABC (ChABC).^13,21,28,38,42–49^ ChABC is routinely used for degrading the chondroitin sulfated GAG chains away from their resident core proteins and has been shown in various models of SCI to have pre-clinical benefits with improvements in both locomotor ability and increased axonal regeneration/sprouting toward and into previously denervated spinal sensory or motor centers.^11,13,20,47,50,51^ There has only been limited success when ChABC was used alone or when coupled with other strategies, such as rehabilitative training, in sub-chronic SCI models. Somewhat improved forepaw function has been reported when ChABC was combined with pellet reaching training when the treatment was given at 4 weeks after dorsal hemi-section injury.^51^ In another study, ChABC combined with lengthy treadmill training beginning 6 weeks after severe contusion cord lesion modestly improved locomotor behavior.^50^ When ChABC was locally delivered over an extended period and combined with neural precursor cells or trophic factors, limited improvements in locomotor function have been reported in clip compression lesioned SCI animals with a 6-week delay prior to treatment.^30^ Unlike in acute SCI, in the chronic state, dense glial scarring has formed around the lesion epicenter, and increases in CSPGs in the PNNs have maximized within deafferented spinal levels.^7,11,15,52^ Further, the inflammatory response has largely died down and the opportunity for neuroprotection has long past.^10^

We had been focusing on the return of diaphragm function at much longer chronic stages after lateral cervical level two hemi-section (LC2H). Recently, we documented some rather remarkable results showing complete and persistent return of diaphragm function at greatly protracted chronic stages (up to 1.5 years post-lesion) upon local matrix and PNN degradation with ChABC placed directly in the vicinity of the denervated phrenic motor pool.^53^ The robust recovery of breathing, which occurs after a near lifetime of paralysis, was surprisingly superior to anything achieved at acute or sub-chronic stages after injury using similar techniques.^54^ The ability to stimulate recovery of such strong, hemi-diaphragm function after enzyme-mediated matrix degradation at C4 began to manifest as soon as 1 month post-hemi-lesion at C2 and, importantly, this repair potential continued to grow over time.^53^ It seems likely that, in partial injury models, improvements via enzyme administration in past studies may have been limited, at least in part because not enough time had been allowed to pass prior to treatment to enable the slow process of potentially meaningful spontaneous plasticity to reach sufficient levels. Although able to breathe with the intact hemi-diaphragm, and to ambulate, feed, and drink, high cervical hemisected animals also exhibit long-lasting deficits in forelimb behavior, including reduced gross and fine movements of the ipsi-lesional forelimb and the paw. In this study, in which animals were subject to injury at C2 and treated after a lengthy time interval at C4, we noted positive changes in gross upper arm function, likely mediated by enzyme diffusion caudally toward the cervical enlargement.^53,55^ Although we had not optimized this treatment for forelimb recovery, we were encouraged by the incidental finding.^53^

The use of ChABC has not been applied clinically because of a variety of potential complications such as heat lability of a bacterial enzyme which, in addition, must be administered intraparenchymally into the injured spinal cord, which can further traumatize an already damaged central nervous system (CNS). Further, the injection field is limited to a discrete area, so positive behavioral changes are dependent on the precision of locally restricted yet functionally relevant alterations in the matrix. Several laboratories have engineered stable, longer-lived ChABC or more widespread viral vector release strategies, although the direct delivery issue remains with these novel approaches.^56–60^ In order to target CSPG mediated inhibition more globally, we focused our attention on the possibility of using systemic agents that could potentially diminish the abundance of CSPGs or disrupt the association with their major receptor without directly touching the spinal cord in the presence of any evolving lesion. To accomplish this, we utilized a high dose of a subcutaneously delivered rPTPσ inhibitor. This Intracellular Sigma Peptide (ISP) is a mimetic of the PTPσ wedge domain that contains a TAT (TransActivator of Transcription) domain that facilitates membrane-penetration. We have previously demonstrated that ISP works acutely *in vitro* and *in vivo* to prevent the conversion of growth cones into a dystrophic state through the excessively tight substrate adhesion mediated via the rPTPσ receptor and the CSPGs.^21,61–63^ In addition, we investigated the therapeutic effectiveness of a clinically approved, orally administered small molecule perineuronal net inhibitor, 4-methylumbelliferone (we call it “perineuronal net inhibitor,” [PNNi], for use in the CNS), which serves to limit a major transmembrane scaffold for PNN assembly, resulting in the PNN being unable to be stably formed or maintained.^64^ Therefore, in addition to using the receptor disrupting wedge peptide (ISP), we used a novel strategy in combination, which can simultaneously and expansively reduce the matrix and PNN CSPGs.^64^

Our chronic LC2H SCI model allows us to test the role of PNN-associated CSPGs in curtailing potential functional sprouting from the intact side of the cord at the level of the cervical enlargement or likely elsewhere within the CNS related to forearm and paw function. By strongly interrupting the rPTPσ–CSPG interaction, we were able to significantly alter the density of the CSPG component of the PNN and restore functional movements of the impaired forelimb during overground locomotor as well as cereal manipulations by the fingers during eating. The cumulative effect of ISP and PNNi further supports the crucial role of CSPG-mediated inhibition via the rPTPσ receptor in curtailing functional synaptic plasticity at lengthy chronic time points following SCI.

## Methods

### Ethical declaration and animal husbandry

All experiments were approved by the Institutional Care and Use Committee at Case Western Reserve University (CWRU), Cleveland. Animals were housed in groups of two or three, and exposed to a normal dark–light cycle with free access to food, water, and environmental enrichment *ad libitum*. The health and welfare of the animals was monitored daily by the study investigators and veterinary staff at Case Western Reserve University.

### Behavioral training and assessments

**FIG. 1.**
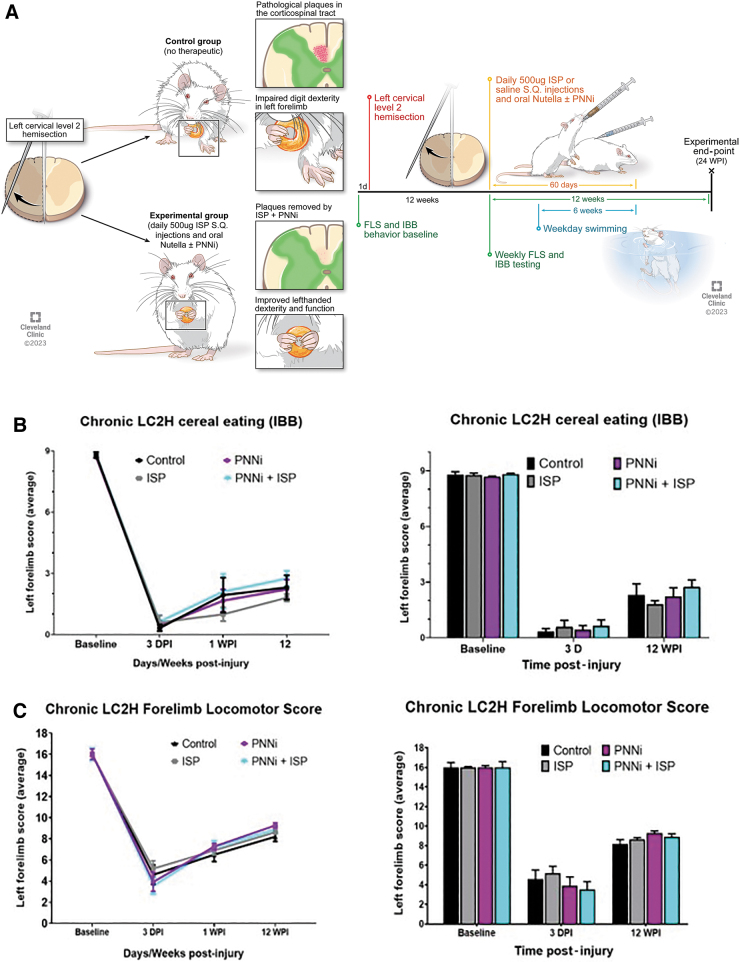
**(A–D)** Incomplete cervical spinal cord injury (SCI) severely impairs forelimb function during walking and eating behavior at chronic stages. **(A)** Timeline of the experimental protocol. Representative image depicting surgical SCI procedure at C2. All animals received a left hemi-lesion at C2 removing ipsilateral descending motor inputs. (**B** and **C**) Lateral cervical level two hemi-section (LC2H) severely impairs forelimb walking ability and cereal eating behavior at 3 days post-injury (DPI), 1 week post-injury (WPI), and 12 WPI. There were no significant differences across the experimental treatment groups compared with controls. Color image is available online.

Animals were acclimated to the laboratory room testing environment and cereals in the home cage for five consecutive days. The animals were also handled by the researchers twice a day to minimize any stress/anxiety prior to exposure to behavioral testing. The following week, the animals were acclimated to the various testing apparatuses, which included a 10-min session each day in the glass cylinder used in the cereal eating assessment. The animals were trained to eat at least three of each cereal type (sphere-shaped: i.e., Cocoa Puffs or donut-shaped: i.e., Fruit Loops) within the 10-min session. For acclimation to the forelimb locomotor testing platform, rats were placed on top of an open field platform ∼10 cm in diameter and allowed to explore for 5 min. Forelimb locomotor and cereal eating pre-training was conducted for five consecutive days. Baseline behavior performance was acquired following training and before LC2H (Fig. 1A).

### Forelimb function assessments

Forelimb function was determined through assessments that involved monitoring behavior that the animals performed naturally. This included the Forelimb Locomotor Scale (FLS)^65^ and the Irvine, Beattie, and Breshnahan (IBB) forelimb recovery scale.^64^ Baseline values were taken prior to injury and at 3 months after injury just prior to daily subcutaneous injection of high dose ISP or saline alone or combined with Nutella ± PNNi oral gavage administration (for 60 days) and then weekly following treatment application (Fig. 1A). Statistical comparisons were made between treatment groups and within behavioral measurements using two-way, repeated-measures analysis of variance (ANOVA) with recommended post-hoc correction (Bonferroni) (GraphPad Prism).

#### Forelimb Locomotor Scale (FLS)

We used established methods detailed by Singh and colleagues to assess forelimb locomotion.^65^ Briefly, rats are encouraged to continuously walk on top of an elevated circular platform ∼10 cm in diameter. Rats that remained stationary for longer than 10–15 sec were enticed to move by having them follow a pencil or a piece of paper, or by lightly tapping or scratching on the side of the open field. If the animal failed to respond to these stimuli, it was picked up by the forequarters and placed in the center of the open field or opposite its previous position, which usually caused it to move. Rats were recorded during locomotion using a high-speed (60 frames/sec [FPS]) camera for offline scoring to assess forelimb walking functionality. Videos were scored in a blinded fashion in slow-motion (at least 50%) playback using the Lightworks video editing software. Recorded videos containing 3–4 min of the animal freely walking on the elevated platform were measured using an 18-point scale (0–17). Animal locomotor behaviors were assessed for impairments and assigned a score reflecting the ability of the animal to perform steps that were consistently plantar, parallel, and weight-bearing.^65^

#### IBB Forelimb Recovery Scale

These testing methods were established previously.^64^ Briefly, animals were placed in the 25 cm H × 20 cm W clear glass cylinder that was used for previous acclimation and training. Each rat was given one sphere-shaped (Cocoa Puff) and one donut-shaped (Fruit Loop) cereal to eat. Their manipulations of the cereals were recorded using a high-speed (60 FPS) camera for offline scoring to measure forelimb and digit abilities during eating. Videos were scored blinded in slow-motion (at least 50%) playback using the Lightworks video editing software. Recorded videos containing the animal eating one sphere-shaped cereal (i.e., Cocoa Puff) and one donut-shaped cereal (Fruit Loop) were measured using a 10-point scale (0–9).^64^ The animal's ability to dexterously manipulate the cereals was assessed for impairments and assigned a score reflecting the ability of the animal to grasp the cereal with subtle adjustments made using the second, third, and fourth digits and wrist movements.^64^

### Stimulation of forelimb utilization

All animals were placed in a pool (4’ diameter) filled to a depth of ∼46 cm at a temperature of 35–38°C to match the rat's natural internal temperature. Animals were placed in the pool for 1 min. Two to four rats were placed in the pool at one time. After swimming, rats were partially dried with a cotton towel and then placed in empty cages lined with paper towels and allowed to further dry and groom themselves uninterrupted for 1 h at room temperature. The rats were then replaced back into their home cages. Swimming took place 5 days a week beginning 2 weeks after treatment application began, excluding the day prior to and the day of behavioral acquisition using the FLS and IBB assessments described (Fig. 1A).

### LC2H injury and systemic treatments

**FIG. 2.**
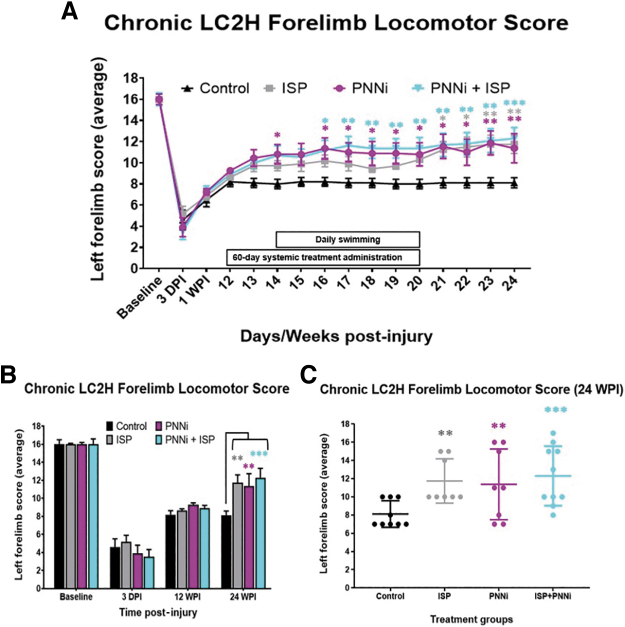
**(A–C)** The Forelimb Locomotor Score (FLS) is significantly improved following systemic treatment to remove chondroitin sulfate proteoglycans (CSPGs) in the perineuronal net (PNN) and/or modulate receptor PTPσ. (**A**) Chronic spinal cord injury (SCI) rats were measured for forelimb locomotor function before and after lateral cervical level two hemi-section (LC2H) and systemic intracellular sigma peptide (ISP) ± perineuronal net inhibitor (PNNi). Both ISP ± PNNi significantly improved weight-bearing stepping after chronic PTPσ-PNN binding perturbation (average FLS score ISP analysis of variance [ANOVA], *p* = 0.037). (**B, C**) At 24 weeks post-injury (WPI), animals receiving either systemic drug showed an average improvement in walking behavior, with combinatory recovery showing the greatest significance and rate of recovery (p* < 0.05, p** < 0.005, p*** < 0.0005). Bonferonni post-hoc analysis, data are reported as average ± standard error of the mean (SEM). Color image is available online.

SCI surgeries were performed as previously described.^53^ Adult female Sprague Dawley rats (280 ± 20 g; Harlan Laboratories Inc., Indianapolis, IN, USA) were anaesthetized with an intraperitoneal injection of ketamine + xylazine cocktail (70 mg kg^−1^/7 mg kg^−1^). The dorsal neck-shoulder area was shaved, cleaned, and sanitized using Betadine and 70% ethyl alcohol, and analgesics were administered through subcutaneous injection of meloxicam (1 mg kg^−1^). Body temperature was maintained and monitored throughout the surgery at 37 ± 1°C. A dorsal midline incision ∼3 cm in length was made over the cervical region. After the skin and paravertebral muscles were retracted, a laminectomy was performed over C2 and the rostral spinal cord was exposed (Fig. 1A). A 21G syringe needle was positioned at the midline of the spinal cord, and a left lateral durotomy and hemi-section were performed caudal to the C2 dorsal roots making certain that the needle tip extended to and was dragged along the ventral bony lamina surface. This process was repeated five times and ranged from the midline to the most lateral extent of the spinal cord. The muscle layers were sutured together with 3-0 Vicryl and the skin was closed using wound clips. The animals were given meloxicam (1 mg kg^−1^) and sterile saline subcutaneously for up to 5 days post-surgery along with nutritional support if their weight dropped >5% of what it had been pre-injury. The anatomical completeness of the injury was confirmed through microscopy and behavioral assessment. Post-injury animals exhibited no signs of malnutrition or infection, but those that exhibited persistent autophagy as a result of the injury before or during the systemic treatment phase were excluded from the study. Before initiating any systemic treatments, all animals were confirmed to have achieved a performance score ≤4.5 ± 1 out of 17 at 3 days post-injury (DPI) as described in the locomotor assessment, and a score of 8 ± 1 12 weeks post-injury (WPI) (Figs. 1C and 2; see Forelimb Locomotor Scale section*)*.^65^ For cereal eating assessments, all animals were confirmed to have received a performance ≤0 ± 1 out of 9 at 3 DPI, and 2 ± 1 at 12 WPI **(**Figs. 1B and 4; see IBB Forelimb Recovery Scale section).^64^

Three months after LC2H, the rats were randomly assigned to begin a systemic treatment protocol that included a daily 0.5 mL subcutaneous injection of 500 μg of intracellular sigma peptide (ISP) under the skin of the back near the lesion, combined with oral gavage feeding of Nutella mixed with sunflower oil with or without the small molecule (0.2 g/mL) perineuronal net inhibitor, PNNi. Experimental animals were fed PNNi mixed with hazelnut spread thinned with sunflower oil twice daily at a dose at 2 g/kg based on weight. Control animals were injected with saline and fed hazelnut spread thinned with sunflower oil not containing PNNi. Two separate cohorts received ISP or PNNi alone. These systemic treatments were administered with the goal of simultaneously disrupting the interaction between would-be sprouting axons and the rPTPσ receptor as well as to reduce the density of inhibitory extracellular perineuronal net components. Each cohort (*n* = 8–10) received the treatments daily for 60 days and were tested and assessed blindly each week for arm and hand function for the duration of treatment, and continuing 4 weeks following the termination of treatments. Our assessments (see details subsequently) of forelimb/paw function were conducted using the FLS^65^ and the IBB forelimb recovery scale.^64^ Both are rating scales similar to the well-known Basso, Beattie and Bresnahan (BBB) scoring method for overground walking, but are focused on the forelimb and digits.^66^

### Low-magnification histology of serotonergic fibers, PNNs, and confirming completeness of the cervical injury

At 24 WPI, animals received an overdose of anesthesia prior to cardiac perfusion with 4% paraformaldehyde dissolved in phosphate buffered saline (PBS). Spinal tissue containing regions of interest were dissected and post-fixed in 4% paraformaldehyde overnight. Prior to sectioning, the tissue was cryoprotected with sequential treatments first in a solution of 30% sucrose in PBS and then in a 1:1 mixture of 30% sucrose in PBS + OCT for 2 days; 30 μm coronal sections of tissue were prepared rostral to caudal from cryoprotected cervical spinal cords (C5–C8) using a Leica Cryostat. Immunofluorescence staining was performed using standard protocols. (Refer to Table 1 for a detailed list of primary antibodies.) In each case, primary antibodies were conjugated to an appropriate Alexa Fluor-containing secondary antibody for microscopic visualization. Nuclei were visualized using Hoechst a 33342 staining. Following the antibody staining, tissue sections were mounted to slides, protected with ProLong Diamond Mounting Medium, cover-slipped, and examined either with an inverted Leica SP8 confocal microscope or a Zeiss Axio Imager microscope.

**Table 1.** Antibodies Used in This Study

Antigen	Host species	Dilution	Vendor	Catalog number
GFAP	Chicken	1/1000	EnCor Biotechnology	CPCA-GFAP
Biotinylated-WFA lectin	-	1/500	Vector Laboratories	B-1355
Cat 301 (ACAN)	Mouse	1/1000	Millipore	MAB5284
SERT	Rabbit	1/1000	EnCor Biotechnology	RPCA-SERT
Iba1	Rabbit	1/1000	EnCor Biotechnology	RPCA-IBA1
Neuro Filament (NF)	Rabbit	1/1000	EnCor Biotechnology	RPCA-NF-H
5 HT	Rabbit	1/1000	Immuno Star	20080
Strepavidin; Alexa Fluor 555	-	1/500	Invitrogen (Life Technologies)	S32355
Mouse; Alexa Fluor 488	Donkey	1/500	Invitrogen (Life Technologies)	A21202
Chicken; Alexa Fluor 488	Donkey	1/500	Jackson Immuno Research	703-545-155
Rabbit; Alexa Fluor 488	Donkey	1/500	Invitrogen (Life Technologies)	A21206
Mouse; Alexa Fluor 555	Donkey	1/500	Invitrogen (Life Technologies)	A31570
Chicken; Alexa Fluor 555	Goat	1/500	Invitrogen (Life Technologies)	A21437
Hoechst 33342, trihydrochloride trihydrate	-	1/3000	Invitrogen (Life Technologies)	A21437

### Quantification of Wisteria floribunda agglutinin (WFA)^+^ aggregates

Tissues from three separate animals per treatment condition were evaluated. For a given animal, 12–15 representative images were captured (∼ 5 images per section per anatomical segment). The number of WFA bright inclusions were totaled using the Cell Counter Plugin of the FIJI Image Analysis Software. In Figure 8P–R, each dot represents the number of inclusions counted in a single image.

### Lesion area and volume

Sixteen spinal cords were selected for analysis from the total available using a random number generator. Sections were obtained using a cryostat at 30 μm/section spanning a ∼15-mm-long cervical segment containing the LC2H. Spinal cord lesion area and volume were analyzed through fluorescent Nissl staining (ThermoFischer) performed on cross-sectioned tissue of each spinal cord at 500-μm intervals. The coverage of the lesion as well as the location was determined using a Zeiss Axio Imager microscope and quantified blindly using National Institutes of Health (NIH) ImageJ software analysis of three to four sections per animal that included the lesion epicenter by using monochromatic image and background readings subtracted at the defined area of interest. In all subjects, the left half of the cervical spinal cord was completely severed from the midline to the lateral-most extent of the tissue, and there were no deviations (Fig, 1A).

### Statistical analysis

For statistical analysis, divergences where *p* < 0.05 were considered significant. Sample size for the *in vivo* behavioral studies was based on a power calculation in which α = 0.05 and β = 0.80, resulting in *n* = 8. Power analysis was conducted prior to all experiments to ensure that numbers (*n*) of animals per group were sufficient. The data are presented as mean ± standard error of the mean (SEM). To ensure a normal distribution, data were subjected to the Shapiro–Wilk test for normalcy prior to analysis. Statistical analysis was performed using a one-way ANOVA with post-hoc Bonferroni correction (GraphPad Prism). At the time of processing and analyses of all experiments and assessments, investigators were blinded as to the treatment group of each animal. The functional and behavioral recordings from every animal in each group were analyzed without exclusion based on the outcome.

## Results

### All treatments improve gross function following a chronic incomplete cervical SCI

All rats displayed a baseline pre-injury FLS performance level at a score of 17 of the (left) forelimb during walking behavior, or consistent plantar stepping of the forepaw, predominant parallel placement. and continuous toe clearing (Figs. 1C and 2A, B). At 3 DPI all rats exhibited an FLS score of ∼4, which improved slightly at 1 WPI to ∼7 (Figs. 1C and 2A).^65^ These findings demonstrate the consistency of the targeted removal of descending supraspinal motor inputs upon inducing the lateral transection across all of the treatment groups immediately following SCI. Unsurprisingly, by 12 WPI, all rats had reached a pre-treatment baseline FLS score of ∼8, indicating that the animals displayed some spontaneous recovery of function that was consistent prior to treatment and not statistically different between groups (Figs. 1C and 2A, B). An FLS score of 8 denotes the animals' ability to perform partial weight-supported steps but only on the dorsal surface of the impaired limb/paw (Figs. 1C and 2A, B and Supplementary Video 1).^65^

**FIG. 3.**
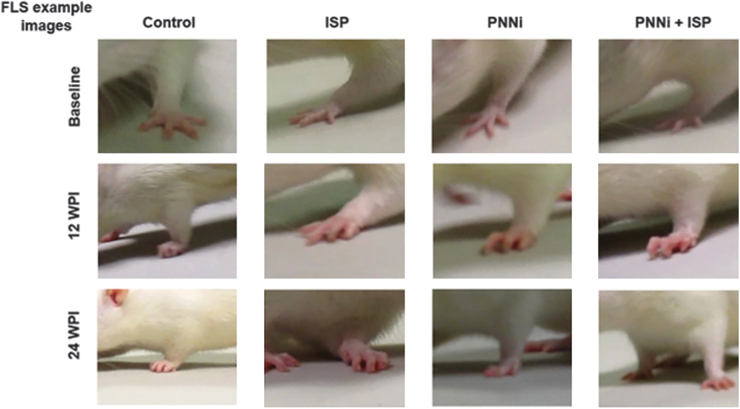
Forelimb Locomotor Scale (FLS) walking assessment. Example still-frame images from actively walking rats at baseline, 12 weeks post-injury (WPI), and 24 WPI (4 weeks after systemic treatment ended) during FLS analysis (refer to Fig. 2 and Supplementary Videos 1–5). Color image is available online.

Interestingly, the control group never gained further FLS assayed function beyond that demonstrated in the pre-treatment assessment, and therefore, any recovery observed is likely a result of the administered treatment (Figs. 2B, C and 3**,** and Supplementary Video 2). The various treatments caused an initial increase in performance as early as the 1st week after their onset (Fig. 2). There was a significant difference between the FLS score at 14 WPI in the animals treated with PNNi alone (Fig. 2A), whereas the combination treatment group revealed a significance in recovery beginning at 16 WPI, which then improved slowly during the drug administration phase (Fig. 2A). At 24 WPI, the control rats maintained an average FLS score of ∼8, whereas rats that received ISP alone, PNNi alone, or both in combination displayed significant improvements in forelimb recovery during walking compared with the control group (Figs. 2 and 3). There was little evidence of synergy between the two treatments. At 24 WPI, animals treated with ISP alone or PNNi alone reached an average FLS score of ∼11, which functionally translates to continuous plantar stepping but with poor wrist control and no toe clearance (Figs. 2 and 3, and Supplementary Videos 3 and 4). The combination- treated rats were slightly better, receiving an average final score of ∼12, indicating continuous weight bearing and plantar stepping that was predominantly parallel but without toe clearance (Supplementary Video 5). However, the best-responding animals in this group could achieve levels as high as 17, which means that during active locomotion, the forelimb stepping performance displayed proper toe clearing and parallel placement of the forepaw, indicating wrist control recovery that was functionality comparable to baseline walking behavior (Fig. 2C).

### Combination treatment best improves skilled forelimb and digit function after a partial chronic cervical SCI

**FIG. 4.**
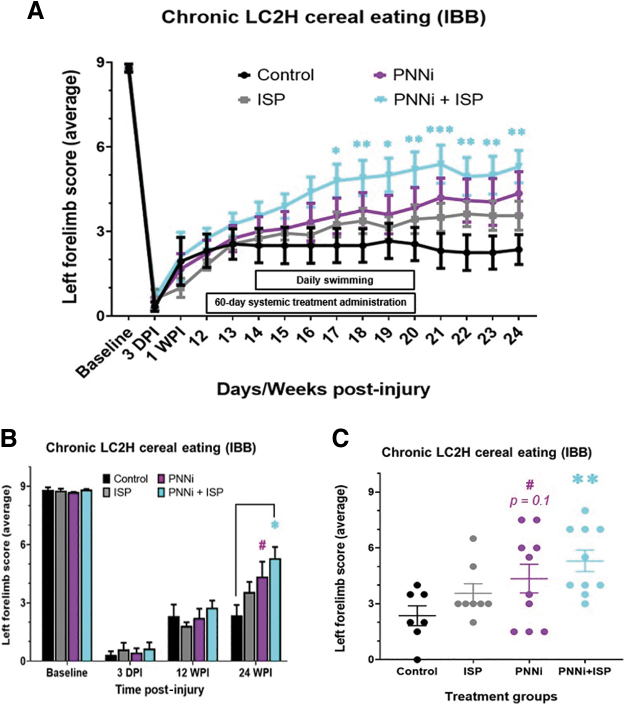
**(A–C)** Cereal eating ability assessed using the Irvine, Beattie, and Breshnahan (IBB) rating scale is significantly improved following combined systemic treatment to both remove chondroitin sulfate proteoglycans (CSPGs) in the perineural net (PNN) and modulate receptor PTPσ**.** (**A**) Chronic spinal cord injury (SCI) rats were measured for forepaw cereal manipulability before and after lateral cervical level two hemi-section (LC2H) and systemic intracellular sigma peptide (ISP) ± PNN inhibitor (PNNi). Only animals receiving combinatory therapy showed significant improvement as early as 17 weeks post-injury (WPI). (**B,C**) Only chronic injured animals receiving ISP + PNNi improved significantly in cereal eating behavior by 24 WPI (panel **C**). Rats that received PNNi alone demonstrated a trend toward significance 4 weeks after the termination of oral administration (p* < 0.05, # = 0.1601). Bonferonni post-hoc analysis, data are reported as average ± standard error of the mean (SEM). Color image is available online.

**FIG. 5.**
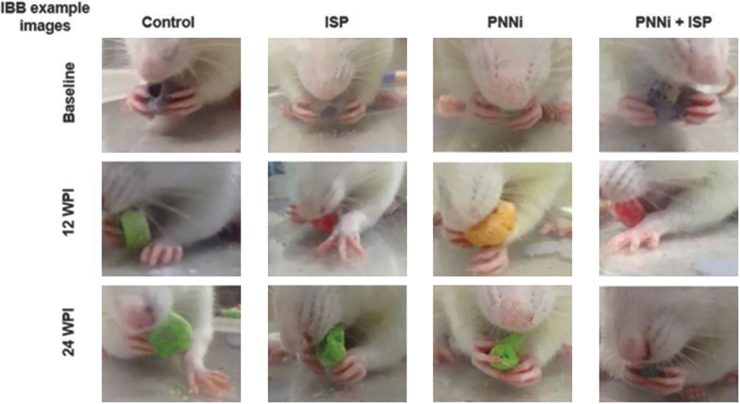
Irvine, Beattie, and Breshnahan (IBB) cereal eating assessment of fine digit and wrist functional recovery. Example still-frame images of animals eating cereal at baseline, 12 weeks post-injury (WPI), and 24 WPI (4 weeks after systemic treatment ended) during IBB analysis (refer to Fig. 4 and Supplementary Videos 6 and 7). Color image is available online.

When assessing for cereal-eating ability, all rats displayed a baseline pre-injury performance level at a score of 9 when both the soon to be injured (left) and uninjured (right) were measured. Normal performance is described as almost always maintaining properly shaped paws (conforming to that of the cereal) during grasping of the cereal with a fully flexed elbow, extensive distal limb movements, subtle cereal adjustments, and volar supported manipulations.^64^ At all weekly behavioral testing time points before and during systemic treatment administration, the uninjured forelimb displayed consistent normal function during cereal eating. At 3 DPI and 1 WPI, all rats exhibited an IBB score of ∼0.5 and ∼2, respectively (Figs. 1B and 5). By 12 WPI, all rats reached a pre-treatment baseline IBB score of ∼2, which translates to cereal-eating behavior that uses the non-volar surface of the injured forelimb, with a predominant fixed forepaw position that is clubbed into a fist-like flexed state, while some animals display an extended forepaw phenotype (Figs. 1B, 4A, B, and 5).^64^ Unlike the FLS data previously detailed, the IBB assay did not reveal a significant effect of treatment until week 4. Further, the significant behavioral recovery observed was only demonstrated in the animals that received both PNNi and ISP in combination (Figs. 4C and 5).

Our behavioral results using the IBB assessment to measure forelimb and paw cereal manipulations further demonstrated that the combination treatment group eventually developed the ability to use their once-paralyzed paw/fingers remarkably better than controls. The control animals recovered paw/finger function only minimally over time (compared with normal animals) to an IBB score between 2 and 3, where the affected paw may remain partially clubbed and mostly rests on the ground and where, at best, some crude, exaggerated cereal adjustments are made (Figs. 5 and Supplementary Video 6).^64^ The paw does not exhibit adaptability and fails to conform to the shape of the piece of cereal. Although animals receiving either ISP or PNNi alone did not show a similar effect of meaningful forelimb functional recovery as we showed in the FLS assessment, these animals did show a strong trend toward significance by the end of the study compared with the control group (Figs. 4 and 5). On the contrary, the combination of ISP and PNNi led to clear improvements in paw/finger function during cereal eating.^64^ On average, at 24 WPI and with the combinatorial treatment, the once- paralyzed animals recovered to an IBB score of between 5 and 6 with extensive contact manipulatory movements (Figs. 4 and 5, and Supplementary Video 7).^64^ Our most encouraging finding was that the best-responding animals, especially when eating a donut-shaped cereal such as a Fruit Loop, could achieve a remarkable score of 7 (9 is the highest possible score)^64^ where sometimes normal, shape-adapting grasping can occur. In summary, these behavioral data confirm the hypothesis that modulating CSPGs in the scar and especially in the PNN as well as in their receptor simultaneously is advantageous to the recovery of precision digit function long after SCI.

### Histological analyses reveal reductions in WFA^+^ matrix and PNNs, and sprouting of serotonin (5-HT) axons in the spinal cord of treated animals

**FIG. 6.**
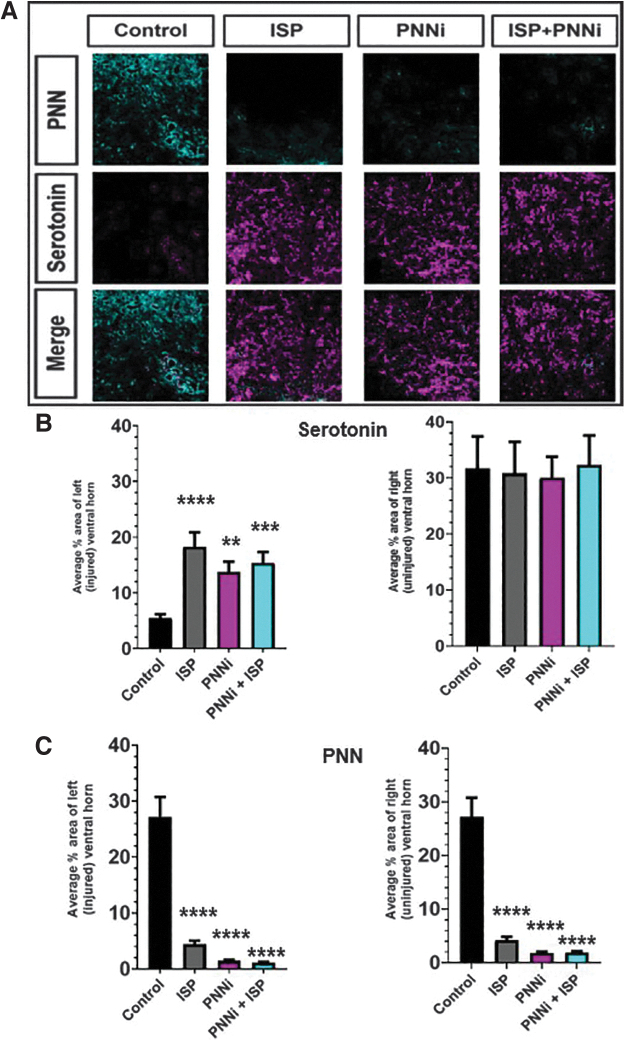
**(A–C)** Perineural nets (PNNs) are decreased and serotonergic fibers are significantly increased in the cervical enlargement gray matter. (**A**) Example immunofluorescent image of sprouted ventral horn serotonergic fibers (magenta) immediately caudal to the lateral cervical level two hemi-section (LC2H) in an animal treated with combined intracellular sigma peptide (ISP) ± PNN inhibitor (PNNi). (**B, C**) Quanitifications of analyzed images shown in (**A**). Serotonergic fibers are significantly increased, while PNNs demonstrate an inversely correlated decrease within the extracellular gray matter of the cervical enlargement. These results are not observed when chronic cervical spinal cord injury (SCI) rats receive saline + Nutella as control (analysis of variance [ANOVA] serotonin [5-HT]: ISP ipsilateral *p* < 0.0001, contralateral *p* = 0.991; PNNi ipsilateral *p* < 0.0033, contralateral *p* = 0.9898; ISP+PNNi ipsilateral *p* < 0.0004, ISP+PNNi contralateral *p* = 0.9993); (ANOVA Wisteria floribunda agglutinin [WFA], ISP ipsilateral *p* < 0.0001, contralateral *p* < 0.0001; PNNi ipsilateral *p* < 0.0001, contralateral *p* < 0.0001; ISP+PNNi ipsilateral *p* < 0.0001, contralateral *p* < 0.0001) Bonferonni post-hoc analysis, data are reported as average ± standard error of the mean (SEM). Color image is available online.

At the end of our behavioral study (24 WPI), we visualized WFA immunoreactivity using WFA staining, focusing on the ventral horn gray matter at spinal levels caudal to the LC2H near the forelimb muscle innervating motor pools for all experimental groups. In control rats that were chronically injured at C2 and received no systemic treatment, there was an upregulation in the accumulation of WFA in the extracellular matrix (ECM) compartment and in PNNs surrounding cells in the cervical enlargement, compared with animals that received ISP with or without oral PNNi (Fig. 6). Incidentally, because WFA-lectin binds numerous glycoproteins, we verified our WFA staining with a well-known aggrecan (*ACAN*)-specific antibody, CAT301. We observed a nearly 1:1 correlation between the staining patterns revealed by CAT301 and WFA, indicating that the WFA staining does represent CSPGs, at least in part (Fig. S1). Although this does not rule out the possibility that the WFA-Lectin has bound other glycoproteins, we can definitively report that CSPGs are contained within the perineuronal net and visualized by WFA. This increased WFA signal was observed throughout the entire cervical region (C5–C8) and was seen on both the injured and uninjured sides suggesting, as have others,^13,15,19,67,68^ that much of the spinal column distal to the lesion responds to a very localized SCI by upregulating the production of CSPGs over a wide distance.

Additionally, the increased inhibitory matrix persists for months (24 WPI) after the initial spinal insult has occurred (Fig. 6). Therefore, in the chronic stage of SCI, CSPGs within the ECM and PNNs are ideal targets for restoration of forelimb movements. Along with the increase in the PNN and extracellular matrix, there was an obvious decrease in the presence of 5-HT^+^ fibers within the cervical ventral horn gray matter (Fig. 6A, B). Together, these data suggest that the observed behavioral deficits following chronic LC2H correlate with increased PNN-CSPGs and loss of descending motor input, including serotonergic axon innervation onto downstream targets. We then compared the WFA matrix and 5-HT^+^ fiber densities in animals that received ISP ± PNNi to the control animals described. In animals receiving daily monotherapy with either ISP or PNNi, a significant decrease of CSPGs in the ECM and PNNs on the injured (left) and uninjured side of the spinal cord was observed (Fig. 6C). A similar (but not beyond that which occurred in the single-treatment animals) reduction in the inhibitory extracellular matrix happened again when both systemic treatments were administered together during the chronic SCI phase (Fig. 6C).

**FIG. 7.**
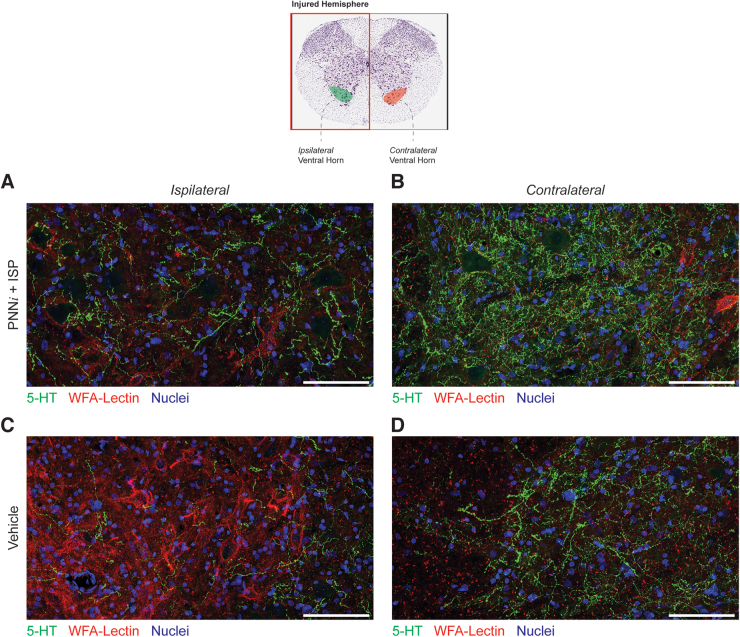
High-magnification histology confirms that perineural net inhibitor (PNNi) + intracellular sigma peptide (ISP) enhances serotonergic innervation. This analysis verified decreased chondroitin sulfate proteoglycans (CSPGs) and increased serotonergic sprouting within the injured ventral horn resulting from daily treatment with PNNi + ISP when compared with the injured, ipsilateral hemisphere (**A, C**). Representative immuno-micrographs demonstrate protection or possible enhancement of serotonergic, axon innervation contralateral to the injury (**B, D**). Scale bar = 100 μm. Color image is available online.

In addition, we measured the serotonergic axon innervation density in the same region. We found that animals that received a systemic treatment daily, either alone or in combination, exhibited a significant increase in 5HT+ fibers within the cervical enlargement compared with animals with unperturbed PNNs (Fig. 6B). Further, at this level of examination, we did not observe a difference in the serotonergic innervation on the contralateral, uninjured side (Fig. 6B). To complement these quantification studies, we examined serotonergic axon innervation at C5 at a higher resolution within the ventral horn gray matter in animals that received daily treatment with PNNi + ISP as compared with controls. We were encouraged to see that these high-magnification studies confirmed our quantified results. We verified decreased serotonergic innervation in the ipsilateral, injured side regardless of treatment condition when compared with the contralateral, uninjured side (compare Fig. 7A and C to 7B and D). Comparing the ipsilateral side of drug-treated versus the vehicle-treated animals, we were able to visualize enhanced serotonergic axon sprouting in the injured, ventral horn resulting from combination treatment with PNNi + ISP. In our previous studies showing the therapeutic efficacy of ISP acutely after severe contusive spinal cord injury,^7^ we demonstrated a rather unique pattern of sprouting of serotonergic axons that occurred in unusually shaped, dense clusters. Upon examination of 5-HT axons caudal to the chronic injury site in our combination-treated animals, we rarely observed this type of clustered axonal sprouting.^7,55,69–72^

### Identification of a novel WFA-Binding Aggregate in the chronically injured cord

**FIG. 8.**
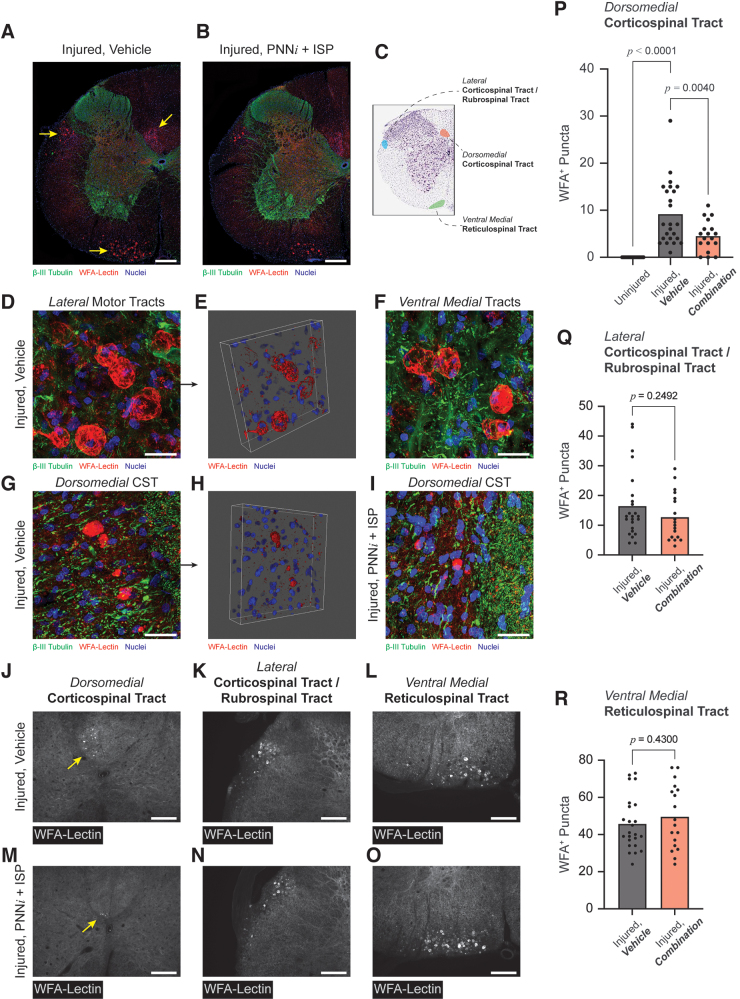
Clearing of chondroitin sulfate proteoglycans (CSPG) aggregates from the dorsomedial corticospinal tract (CST) is associated with behavioral recovery**.** At 24 weeks post-injury (WPI), low-magnification immuno-microscopy (**A, B**) reveals that CSPG aggregates are isolated to three descending motor pathways: (1) the dorsomedial CST, (2) the rubrospinal tract or possibly the lateral CST, and (3) the ventral medial reticulospinal tract (**C**). Aggregates with the lateral and ventral medial pathways were large diameter, globular structures (**D–F**). In contrast, aggregates within the dorsomedial CST were smaller in diameter and presented ellipsoid or spherical morphologies (**G–H**). Quantification of CSPG aggregates from these three anatomical sites (**J–O**) revealed that treatment with perineuronal net inhibitor (PNNi) + intracellular sigma peptide (ISP) associated with substantial clearing of these plaque-like structures from the dorsomedial CST (**P**). Aggregates within the lateral and ventral medial pathways remained intact (**Q, R**). **A, B** scale bar = 250 μm; **D–I** scale bar = 25 μm; **J–O** scale bar = 100 μm. *P* value for panel **P** was determined based on one-way analysis of variance (ANOVA); *p* values for panels **Q** and **R** were determined by unpaired *t* test. Color image is available online.

Our efforts to elucidate the neuronal mechanism of recovery from chronic injury led us to a secondary, never-before-identified phenomenon that was tightly correlated with behavioral improvements. Throughout the cervical enlargement, from the level of injury at C2–C9, we noted numerous, roughly spherical, aggregations of matrix. These plaque-like structures, which we never observed in the cervical cords of age-matched, uninjured animals, stained intensely with WFA-lectin and presented largely within the lesioned white matter where they were localized to a small subset of descending tracts: (1) the dorsomedial corticospinal tract (CST), (2) the rubrospinal or possibly the lateral CST, and (3) the ventral medial reticulospinal tract (Fig. 8A–C). These structures ranged in size from ∼25^_^40 μm in diameter within the lateral and ventral medial tracts (Fig. 8D–F) to 5–10 μm within the dorsomedial CST (Fig, 8G–I). Although these structures were reliably identified using WFA-lectin, they may be composed of only select CSPGs. For example, they could not be visualized using a CAT-301, aggrecan-specific antibody (data not shown).

The precise mechanisms of action of PNNi plus ISP remains unknown. The recovery we observed from treatment with this combination may involve the generalized loss of extracellular CSPGs and overall increase in 5-HT sprouting visualized in Figures 6 and 7. However, these observations cannot account for the superior recovery of digital dexterity resulting from combination treatment over the individual compounds reported in Figure 4. Remarkably, in the ISP + PNNi combination group, we noted substantial clearing of these WFA^+^ aggregates exclusively from the midline CST (Fig. 8J, M, and P). Surprisingly, these structures were not cleared from the other motor pathways in the chronically injured spinal cord (Fig. 8K, L, N, O, Q, and R). Although we do not yet understand what these structures are or from where and when they emanate or why their enhanced clearance is restricted to the midline CST, it is striking that their removal strongly correlated with recovered forelimb function and digital dexterity.

### Matrix aggregates are internalized by microglia in the dorsomedial CST

**FIG. 9.**
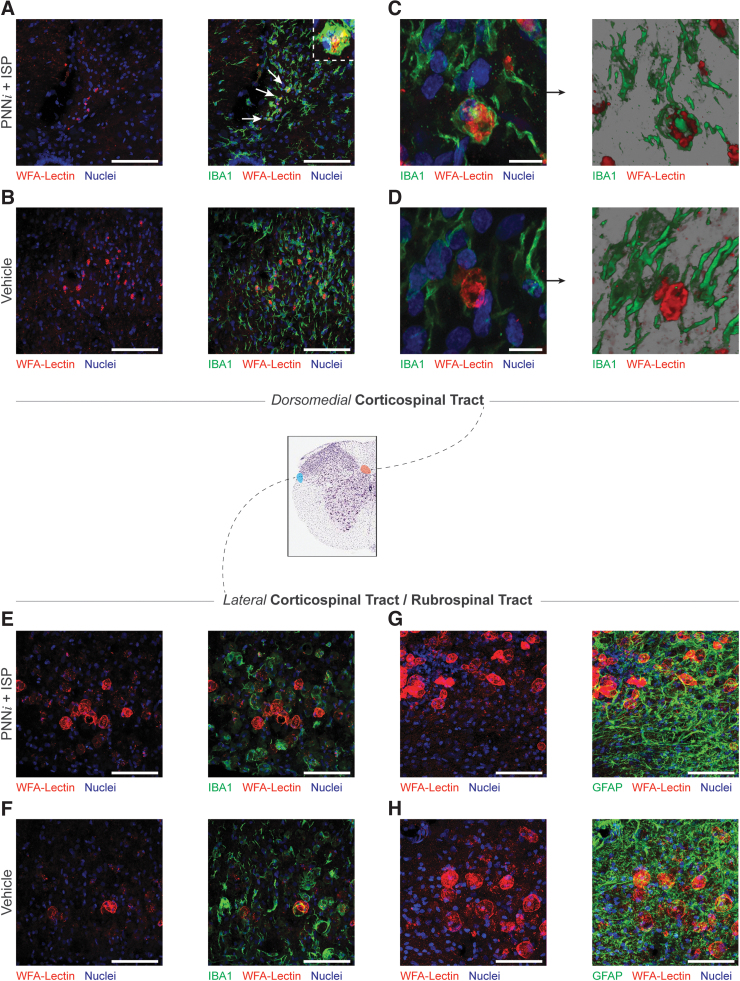
Microglia may clear chondroitin sulfate proteoglycan (CSPG) aggregates from the dorsomedial corticospinal tract (CST). Histological analysis of the cervical cord (C5–C9; 24 weeks post-injury) revealed that IBA1^+^ microglia internalize CSPG aggregates within the CST of combination-treated animals (**A–D**). Internalized aggregates are identified with white arrows in panel **A**. The larger diameter aggregates in the lateral motor tracts are proximal to microglia but were neither internalized nor cleared in either condition (**E, F**). CSPGs accumulated within fields of reactive astrocytes (**G, H**), especially in lateral motor tracts; however, it was not clear whether reactive astroglia were engaged with the generation or clearance of these structures. Scale bar = 100 μm. Color image is available online.

Having identified these pathological WFA^+^ structures and correlated their selective removal to precision forelimb and fine digit motor recovery, we sought a deeper understanding of the neural cells with which they were most closely associated. Using immunofluorescence, we interrogated the microenvironment of the chronically injured cervical cord in animals treated with PNNi plus ISP compared with controls. We examined the spatial relationship of aggregates to microglia using allograft inflammatory factor 1 (IBA1, Fig. 9A–F) as well as to reactive astrocytes using glial fibrillary acidic protein (GFAP, Fig. 9G, H). Although aggregates presented within fields of reactive astrocytes, it did not appear as though these cell types were actively engaged in the generation, clearance, or encapsulation of these structures. In contrast, in the combination-treated animals, microglia were intimately associated with them. In the dorsomedial CST only, microglia/macrophages appear to have internalized the WFA^+^ spheroids, suggesting that clearance may involve inflammatory cell-mediated phagocytosis. Note the co-localization of WFA and IBA1 identified by white arrows and the insert in Figure 9A. In total, these data suggest the possibility that matrix aggregates containing CSPGs represent a newly identified, pathological structure that presents in the chronically injured spinal cord. Additionally, the combined use of PNNi and ISP may alter the physiological state of the microglia which, in turn, facilitates phagocytic removal of the aggregates.

## Discussion

We assessed improvements of forelimb behavior in a hemi-section model of chronic cervical SCI and have revealed that treatments with PNNi and ISP alone but especially in combination can stimulate a good measure of recovery. Further, these functional improvements in the forelimb persisted for at least 4 weeks after the daily treatment(s) had ended. When given for 2 months following a chronic LC2H, ISP or PNNi administered alone were able to improve proximal and distal forelimb use during more crude locomotion. Moreover, we assessed fine motor behaviors during cereal eating and noted rather remarkably improved digit function when we administered our systemic treatments together.

It is quite interesting that recovery of forearm function begins to occur relatively rapidly following systemic treatment in our chronic injury model. Surprisingly, behavioral improvements occur much more quickly than they do after similar treatments during the acute stage.^14^ This time frame mimics that which we have shown previously in our ChABC-treated chronic LC2H SCI respiratory model suggesting, again, that sprouting likely had already occurred but was somehow being masked by the CSPG component of the PNN.^53^ Thus, in our 12-week cervical hemi-lesion model, certain sub-populations of the contralateral descending supraspinal tracts with small numbers of axons that re-decussate within the spinal cord caudal to the level of injury,^72^ or intervening interneurons with similar properties of plasticity,^73^ may be very slowly sprouting new processes that re-cross the midline to the denervated side ipsilateral to the lesion even in the presence of the PNN.^74–78^ Of course, we are not ruling out the possibility that this process may be augmented during the further 2 months of treatment. Various motor systems underlying forelimb and paw movement, including the serotonergic, propriospinal, and even the cortico-spinal neurons have an innate propensity to sprout on their own, although at a very diminished rate.^44,72,73,78–81^ These new collaterals could produce latent, or inactive synapses which, over time, could be beneficial, harmless, or possibly even disadvantageous to recovery.^53,82^ Our results demonstrate that the potential activity of such sprouting can be supportive to functional recovery but is largely kept dormant by CSPGs within the PNN in the vicinity of the relevant motor pools.

There is a question as to what is the mechanism by which the PNN may curtail synaptic activity and perhaps help to create such silent synapses. rPTPσ might be more directly involved with the formation, maintenance, or function of these new connections. Increases in PNN density and changes in the chondroitin sulfation pattern to a more growth-inhibitory state occur during normal aging^83^ and after SCI.^16,84^ It is well established that the PNN plays important roles not only in regulating various morphological aspects of axonal sprouting during the development of critical periods^4,16,18^ as well as after injury in the adult,^7,15,17,41,47,49,52,85^ but also in the control of synaptic activity.^84,86–89^ Interestingly, in a model of Alzheimer's disease, the PNN in the cortex encroaches into synaptic clefts, which shrinks the perimeter of ECM barriers around the PNN holes, thereby reducing the diameter of the portals for communication between pre- and post-synaptic elements.^90,91^ However, although the function dampening consequnces of peri-neuronal net invasion of the synapse has been suggested, the mechanism of this possible physiological barrier property has not yet been fully elucidated.^91,92^ Latent synapses are known to occur throughout the CNS.^93^ One of the most interesting and well-known latent descending motor projections is the crossed phrenic pathway.^77,94–96^ It is already present but can be revived in the adult in seconds after an LC2H injury by creating anoxic stress after lesioning the contralateral phrenic nerve^53,77^ as well as by modulating the leukocyte common antigen-related (LAR) family receptors.^97,98^ It can also be reawakened rather rapidly long after cord injury by administration of chondroitinase.^49,53^ Axons of the corticospinal system are known to re-cross the midline of the spinal cord rather extensively during normal development, but such ipsilateral CST projections are largely eliminated.^99^ It is possible that there might be a would-be pruned cohort of double decussated CST axons that actually persists in the adult but is difficult to label because it exists in a withered, quiescent state. Perhaps, in addition to other proposed mechanisms that can lead to synaptic dysfunction, such connections of sprouted or dormant axons in the cervical enlargement could be made inactive via the matrix-invading phenomenon which, in turn, might be reversed by PNNi removal of the net.^94–96,100–103^

There is a question as to what might be the mechanism by which ISP modulation of rPTPσ mediates functional recovery. In addition to its role in curtailing axonal sprouting and/or regeneration via excessively strong adhesion to CSPG substrates,^7,104,105^ there is also abundant evidence, although mostly *in vitro*, that rPTPσ can function in neural circuit assembly by tightening the bond among its multiple ligands at developing synapses.^25,106–111^ Thus, ISP-mediated disruption of the receptor could loosen its connections with a variety of binding partners and allow for axonal sprouting, albeit at the expense of synaptic reformation.^97,98^ Although we did not quantify synaptic densities in the reinnervated portions of the combination-treated spinal cord denervated by the hemi-lesion, we have reported an increase, not a decrease, in penumbral synapses in a functionally beneficial ISP-treated mouse model of malignant stroke.^105^ Importantly, the notion that rPTPσ as well as the other LAR family receptors are critical organizers during synapse formation has become controversial because of work that showed that synaptic connectivity in the hippocampus was completely unaffected by *in-vivo* deletion of all three LAR-rPTPs beginning in the neonate.^89^ However, more recently, clear *in-vivo* evidence has been presented for a role of rPTPδ in the assembly and maintenance of climbing fiber synapses on Purkinje cells in the developing cerebellum.^112^ Therefore, although the precise mechanism by which ISP leads to functionally beneficial sprouting remains elusive, the manner in which it does this may be indirect and downstream of rPTPσ signaling involving the hypersecretion of specific inhibitory matrix degrading proteases (see subsequent discussion).

Recently, an independent study confirmed the benefits of subcutaneous peptide treatment showing that 500 μg/day of ISP given acutely after lateral crush greatly improved hindlimb locomotor, sensory, and bladder function.^113^ Indeed, with the use of the well-characterized BBB locomotor rating scale, the study's contusion-injured animals (that stabilized at a control baseline score of 6) on average recovered by a remarkable 9 full points to a level of 15.^113^ Importantly, although bladder function (but not walking) showed a dose response when cord-injured rats were given ISP at a maximum of 44 μg/day,^7^ the much higher dose of 500 μg/day provides the first evidence for a dose response by ISP in promoting enhancements in locomotor behavior, at least after acute SCI. Similarly, ISP has been shown to have some therapeutic effects after acute T8 cord hemi-section in the adult rat when assessed by an inherently objective automated gait analysis system.^62,114^ ISP has also now been shown to be effective in promoting regeneration and functional recovery after SCI when delivered via a plasma exosome- based biological scaffold^115^ as well as by a self-assembling peptide hydrogel.^116^ The regenerative potential of blocking the rPTPσ receptor by ISP has, in addition, been demonstrated in models of multiple sclerosis (MS),^8^ ischemic and hemorrhagic stroke,^104,105^ ischemic heart attack,^117,118^ and peripheral nerve injury.^119,120^ The high dose of 500 μg/day of ISP is what we chose to use, and was effective in our chronic study. However, the most optimal dosing regimen and the best route of delivery, as well as the optimal amount of peptide that is most functionally beneficial have not yet been established. Also, it is important to stress that as a standalone treatment, ISP is not especially effective in cases of extremely severe cord injury, as our laboratory has recently observed in ongoing studies using a more complete model of thoracic spinal cord injury (data not published).

Hyaluronan (HA) is the major scaffold for PNN assembly, and if HA synthesis is blocked, then the PNN is not able to structure itself properly.^113,121,122^ In addition to the use of the wedge peptide ISP, we employed a novel strategy in combination, which can substantially reduce the PNN CSPGs in the CNS via systemic delivery of an already clinically approved small molecule proteoglycan synthesis inhibitor called 4-methylumbelliferone (4MU) or hymecromone.^121–123^ Referred to as PNNi in our study, we repurposed this small molecule in our model of chronic SCI. One of the major challenges for translation of SCI-regenerative strategies from rodent to human is the enormous relative difference in size of the human, which necessitates careful considerations of concentrations, toxicity, and routes of delivery to optimize the amount and time of residence of the therapeutic drug within the CNS. Oral delivery of a small molecule inhibitor that is already clinically approved is a preferred strategy for drug delivery. A novel therapeutic approach to overcome the inhibitory effects of the PNN involves curtailing the production of HA. PNNi specifically inhibits HA synthesis by acting as a competitive substrate for hyaluronan synthase, an enzyme critical for HA production.^122^ Preliminary findings have shown that inhibiting HA synthesis with PNNi resulted in significant decreases in the hyaluronan link protein as well as in the amount of CSPGs in an *in-vitro* model as well as in the brain and spinal cord. *In vivo*, the reduction of the CSPG component of the PNN was especially prominent in the ventral horn around motor neurons.^124,125^ We therefore hypothesized that PNNi, especially when administered together with ISP, could be an obvious dual therapy, because the combined strategy would decrease the ligand as well as modulating the receptor globally, and could be an effective, clinically viable treatment method following chronic partial cervical lesions. Indeed, we confirmed that chronic disruption of proper PNN assembly in the two cohorts of animals that received 4-MU (± ISP) significantly reduced the presence of WFA+ CSPGs within the cervical enlargement. Importantly, the reduction in CSPGs was inversely correlated with the increased density of serotonergic axons observed within the ventral gray matter of the same region. PNNi as well as ISP are non-invasive treatments with low toxicity that can be stopped at any time to reverse the receptor blockade and HA-synthesis-inhibiting effects of the combination, and allow for the PNN to be re-established. This can help avoid potential long-term adverse side effects and help stabilize newly formed synapses.

Although reduction of CSPGs in the 4-MU-treated animals was expected, there was a question as to why there was also a reduction of CSPG matrix, again where 5-HT axon densities had increased, in the animals treated with ISP only. A variety of motile cell types, including neurons, use tightly regulated release of proteases to remodel the surrounding extracellular matrix along their potential routes of migration.^69,126–128^ We have described, *in vitro* and *in vivo*, an interesting downstream event in both dorsal root ganglion and serotonergic neurons that occurs as a consequence of rPTPσ modulation by ISP, resulting in the locally enhanced release of the matrix-degrading enzyme, cathepsin B. Cathepsin B is a proteolytic enzyme that is normally produced, albeit in limited amounts, by growing neurons during crucial periods of extracellular matrix remodeling, and is also augmented in neurons within the spinal cord gray matter after injury.^129,130^ The induced exaggerated secretion of cathepsin B, likely at the leading edge of ISP-treated axon growth cones, helps them to navigate within or past an inhibitory CSPG barrier.^69,129,130^ We have shown that this phenomenon of enzyme hyper-secretion, which could synergize with PNNi, is protease specific and occurs not only in neurons but also in multiple migratory cell types that bear the rPTPσ receptor as they encounter the CSPGs. Thus, in oligodendrocyte progenitor cells in models of MS as well as subventricular zone (SVZ) derived neural progenitor cells in an ischemic model of stroke, ISP specifically stimulated release of matrix metalloproteinase (MMP)2. The protease encouraged digestion of glial-scar-associated CSPGs which, in turn, restored stem cell migrations and their differentiation through the penumbra and well into the lesion core, resulting in functional improvements.^8,69,104^

Descending serotonergic fibers are known to influence, indirectly or directly, the excitability of spinal cord motoneurons, and it is well established that 5-HT plays an important role in modulating locomotion.^131–133^ Many studies have shown that functional recovery following a SCI is potently influenced by increased numbers of serotonergic axons at spinal levels caudal to the initial injury.^7,53,69^ Whether the increased 5-HT fiber density, that we have described here, plays a role in restoring fine digit control is not known, but there are several studies that have shown a role for 5-HT in the modulation of certain aspects of distal flexor function.^70,71^

Although we did not observe enhanced dorsal CST sprouting or frank regeneration through the lesion (the CST is a tract well known for its role in voluntary control of distal musculature)^134–138^ we did observe a rather dramatic change in the inflammatory reaction to the large plaque-like CSPG deposits only in the lesioned white matter of the dorsomedial CST. It is now known that ISP can promote a more M2-like, reparative immune response after SCI.^139^ Conceivably, this immune-system-related CSPG clearing phenomenon in the dorsal white matter might be occurring at some level far more broadly, even within the gray matter distal to the lesion, suggesting that such activated microglia could also be playing a role in the local elimination of the PNN in the spinal cord leading to increased synaptic plasticity from the undamaged portions of the CST as well as the serotonergic system. However, whether this drug-induced, phagocytic reaction in the degenerating portions of the dorsal CST is a reflection of a wound-healing contingent of microglia throughout the entire corticospinal system is unknown. Therefore, the anatomical substrate (whether it be newly sprouted or latent) that underlies such robust functional recovery chronically as well as the origin and biological impact of the WFA+ plaques must await further studies.

## Transparency, Rigor, and Reproducibility Summary

The study was registered after the study began. The study is registered at BioRxiv (https://www.biorxiv.org/content/10.1101/2022.08.01.502398v).^1^ The analysis plan was not formally pre-registered, but the team member with primary responsibility for the analysis certifies that the analysis plan was pre-specified.^2^ A sample size of eight subjects per group was planned based on an expected effect size calculated to yield 88–96% power to detect significant behavioral recovery using repeated measures ANOVA with a *p* value of <0.05.^3^ Sixty animals were considered for inclusion; 10 animals per group (4 groups) for SCI, behavior, and histological analysis; 32 animals received each treatment; 10 animals died from post-operational complications; and 11 animals had incomplete assessments and were excluded from the study.^4^ Subjects were randomly assigned to groups using a random number generator.^5^ Investigators who administered the therapeutic intervention were blinded to group assignment by use of an identically appearing placebo treatment. Investigators who conducted the outcome assessments were blinded to group assignment by restricting contact with investigators who administered the therapeutic intervention.^6^ Timing of administration relative to injury was 12 WPI. Purity of pharmacological reagents was 99% based on certificate of analysis. Controls used included naïve, uninjured animals and vehicle-only injured animals. Specificity and anatomical accuracy of lesions used for experimental manipulations were verified using fluorescent Nissl.^7^ All the materials used for the therapeutic intervention came from a single batch prepared on May 4, 2020 at Case Western Reserve University School of Medicine's Neuroscience Department.^8^ The experimental injury model is an established standard in the field. The primary outcome measure is an established standard in the field. Validation is included (https://doi.org/10.3389/fneur.2014.00116; https://doi.org/10.1016/j.jneumeth.2014.01.001).^9^ The statistical tests used were based on the assumptions of normal distributions and the sample sizes reflect the number of independent measurements. Non-independent measurements have been addressed using two-way, repeated measures ANOVA.^10^ Correction for multiple comparisons was performed using Bonferroni correction.^11^ Planned/ongoing external validation studies have been pre-registered at BioRxiv.^12^ There is no analytical code associated with this study.^13^ All materials used to conduct the study are available to qualified investigators via direct request from the corresponding author, and purchase from the GoldBio vendor.^14^ The authors agree to publish the manuscript using the Mary Ann Liebert Inc. “Open Access” option under the appropriate license.^15^

## Supplementary Material

Supplemental data

Supplemental data

Supplemental data

Supplemental data

Supplemental data

Supplemental data

Supplemental data

Supplemental data
