# Supplementary figures and images for: Recovery of Forearm and Fine Digit Function After Chronic Spinal Cord Injury by Simultaneous Blockade of Inhibitory Matrix Chondroitin Sulfate Proteoglycan Production and the Receptor PTPσ
Source: J Neurotrauma. 2023 Nov 30;40(23-24):2500–21. doi: 10.1089/neu.2023.0117 (PMC10698859; doi:10.1089/neu.2023.0117)

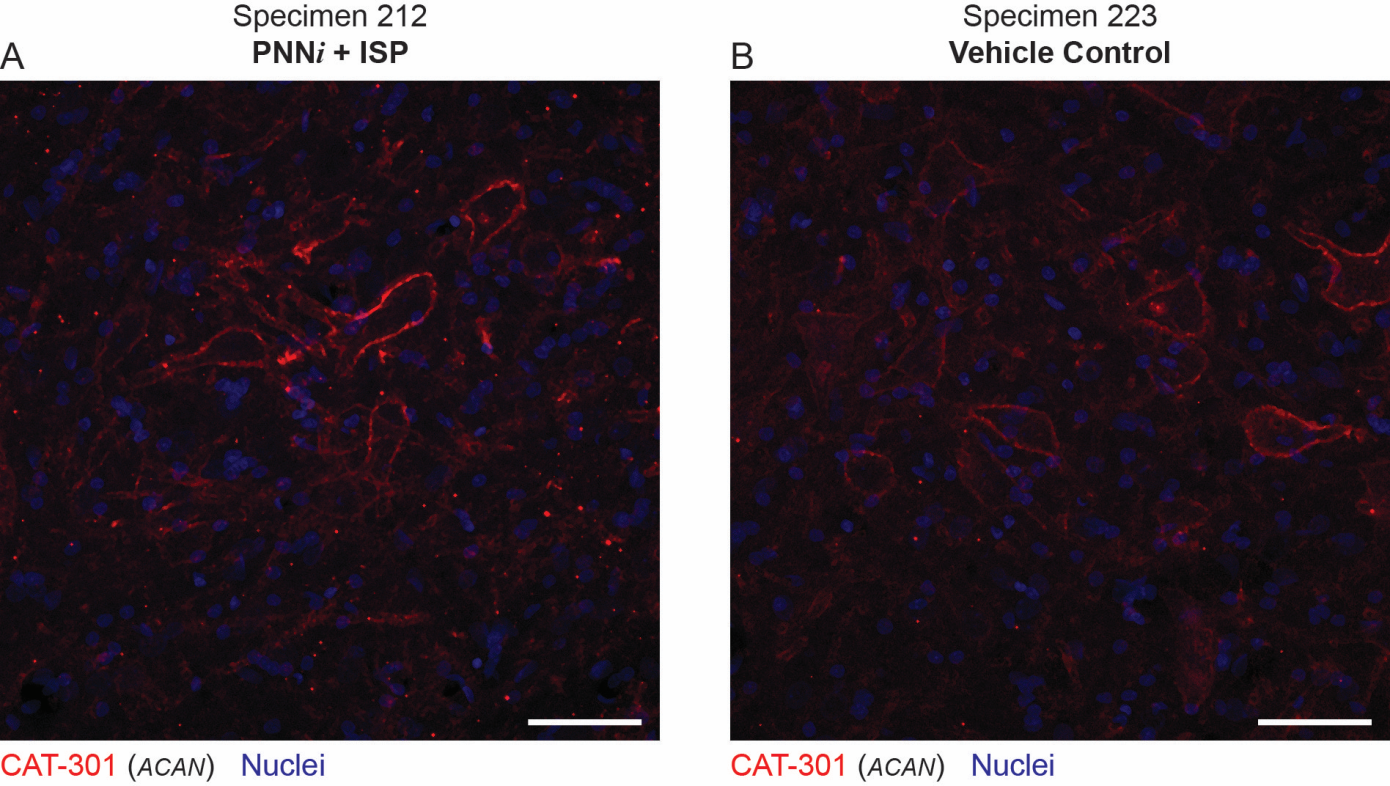

Supplement: Supplemental data [file Suppl_FigS1.docx]
